# *In Vivo* Voltage-Sensitive Dye Study of Lateral Spreading of Cortical Activity in Mouse Primary Visual Cortex Induced by a Current Impulse

**DOI:** 10.1371/journal.pone.0133853

**Published:** 2015-07-31

**Authors:** Tamás Dávid Fehérvári, Yuka Okazaki, Hajime Sawai, Tetsuya Yagi

**Affiliations:** 1 Division of Electrical, Electronic and Information Engineering, Graduate School of Engineering, Osaka University, Suita, Osaka, Japan; 2 Center for Advanced Medical Engineering and Informatics, Osaka University, Suita, Osaka, Japan; 3 Division of Medicine, Graduate School of Medicine, Osaka University, Suita, Osaka, Japan; University College London, UNITED KINGDOM

## Abstract

In the mammalian primary visual cortex (V1), lateral spreading of excitatory potentials is believed to be involved in spatial integrative functions, but the underlying cortical mechanism is not well understood. Visually-evoked population-level responses have been shown to propagate beyond the V1 initial activation site in mouse, similar to higher mammals. Visually-evoked responses are, however, affected by neuronal circuits prior to V1 (retina, LGN), making the separate analysis of V1 difficult. Intracortical stimulation eliminates these initial processing steps. We used in vivo RH1691 voltage-sensitive dye (VSD) imaging and intracortical microstimulation in adult C57BL/6 mice to elucidate the spatiotemporal properties of population-level signal spreading in V1 cortical circuits. The evoked response was qualitatively similar to that measured in single-cell electrophysiological experiments in rodents: a fast transient fluorescence peak followed by a fast and a slow decrease or hyperpolarization, similar to EPSP and fast and slow IPSPs in single cells. The early cortical response expanded at speeds commensurate with long horizontal projections (at 5% of the peak maximum, 0.08–0.15 m/s) however, the bulk of the VSD signal propagated slowly (at half-peak maximum, 0.05–0.08 m/s) suggesting an important role of regenerative multisynaptic transmission through short horizontal connections in V1 spatial integrative functions. We also found a tendency for a widespread and fast cortical response suppression in V1, which was eliminated by GABA_A_-antagonists gabazine and bicuculline methiodide. Our results help understand the neuronal circuitry involved in lateral spreading in V1.

## Introduction

In the primary visual cortex (V1), many neurons can be activated by visual stimuli applied outside the boundaries of their classical receptive field (RF) [1, 2]. Imaging with voltage-sensitive dye (VSD), which is mainly sensitive to subthreshold synaptic potentials [3], observed that cortical activity spread horizontally beyond its retinotopic initiation site [4–9].

Elucidating the spatiotemporal properties of the horizontal propagation of excitatory signals, either sub- or super-threshold, is important to understand the function of V1 in visual perception, since it might play a crucial role in visual processing, such as in improving the specificity and reliability of visual responses [10], in formation of dynamic activity patterns relevant to illusions [5, 9, 11, 12]. It could also play a role in context dependent response tuning from non-classical RF [13–17], since it would activate not only excitatory neurons but also inhibitory interneurons.

In recent works, the mouse is frequently used in anatomical and physiological approaches to study the visual system of mammals. The structure and function of V1 neuronal circuitry relevant to horizontal propagation have been investigated on multiple levels in rodents, including the mouse. On the structural level, anatomical studies have extensively discussed horizontal projections in rat V1 [18–20], which are believed to be present in the mouse as well. The spatial organization and temporal dynamics of signal propagation in V1 circuits have been studied by electrical stimulation of the neuronal networks of cortical slices of rodents: rat [21–25], guinea pig [26] and mouse [27, 28]. These works provided essential information to the understanding of horizontal propagation in intact neuronal circuits *in vitro*. Horizontal propagation in mouse V1 has also been described *in vivo* in response to visual stimuli [29]. Visual stimulation is used in most *in vivo* studies as it evokes responses through the natural pathway of visual processing. This, however, has its drawbacks when one tries to investigate how V1 neuronal circuitry shapes the spatiotemporal properties of cortical activity, since the visually evoked cortical responses are strongly affected by the state of visual circuits prior to V1, i.e. retina and LGN.

Measurement of signal propagation induced by intracortical electrical stimulation *in vivo* is useful in the investigation of V1 neuronal circuitry, linking experiments in slice with visual stimulation *in vivo*. Direct intracortical stimulation removes the additional complexity caused by initial processing in processing circuits prior to V1. Furthermore, the cortical response to electrical stimuli *in vivo* can be studied in reference to synaptic and spike activities induced by electrical shocks in slice preparations in rodents [22, 23, 30–34].

In their 1993 study, Orbach and Van Essen [35] used this approach together with VSD imaging to examine evoked cortical responses in rat visual cortex, and they demonstrated the horizontal spreading of the signal in V1. Such analysis has not been reported in the mouse, however. With advances in technology since their study it is also possible to provide a more detailed quantitative description of the propagation. In the present study we used intracortical electrical stimulation combined with fast VSD imaging *in vivo* to examine how V1 neuronal circuits contribute to the dynamics of horizontal propagation in mouse. We applied single-pulse electrical stimuli to layer 2/3 in V1 of anesthetized mice and recorded the evoked cortical activity at a high spatial and temporal resolution (100×100 pixels, 1 ms/frame). We describe quantitatively the spatiotemporal properties of the propagating response. These results help us understand the V1 cortical network involved in horizontal propagation from a perspective that links slice and visual stimulation experiments.

## Materials and Methods

### Ethics Statement

All experiments were approved by the Institutional Animal Care and Use Committee of the Graduate School of Engineering, Osaka University (permit number 17-6-0), and were conducted in accordance with the Guiding Principles for the Care and Use of Animals in the Field of Physiological Sciences of the Physiological Society of Japan and Guidelines for Animal Experiments of Osaka University. All surgery and recording was performed under urethane anesthesia, and all efforts were made to minimize suffering. The level of anesthesia was assessed by pinching and additional small doses of urethane were injected when necessary. The animals were sacrificed by decapitation in deep anesthesia after administration of a large dose of the anesthetic.

### Animals

Experiments were performed on 17 adult C57BL/6 mice (8–20 weeks; CLEA Japan, Inc., Tokyo, Japan). One additional mouse was used to verify electrode locations only. The mice were kept in a room under a 12-h light/dark cycle and provided food and water ad libitum.

### Surgery and VSD staining

Anesthesia was induced with intraperitoneal (i.p.) injection of urethane (1.25 g/kg body weight) and further small doses were added when necessary to maintain level of anesthesia. Atropine (0.01 mg, i.p.) and dexamethasone (0.02 mg, i.p.) were administered to suppress mucus secretion and brain oedema, respectively. The skin was shaved and treated with a local anesthetic (Xylocaine 20 mg/mL, AstraZeneca) before making incisions. After tracheotomy and cannulation, the animal was placed in a stereotaxic apparatus (SR-15; Narishige, Tokyo, Japan). Body temperature was maintained at 36.8°C with a heating pad, and a gentle flow of oxygen was directed at the mouth of the animal. Craniotomy was performed on the right posterior parietal bone to expose the visual cortex (0.5–4 mm from the midline and 0–3.5 mm from the right lambdoid suture). The dura was left intact. To create a chamber above the exposed cortex, a ~1-mm length silicone-rubber tube (inner diameter, 6.5 mm) was attached to the right posterior parietal bone using dental resin. Bleeding from the dura was stopped completely and the dura was dried thoroughly to increase its permeability [36]. Staining was performed by bath application of RH1691 (Optical Imaging, Rehovot, Israel), at the optimal concentration (1 mg/mL in saline with 1.96 U/mL heparin and 0.125% dimethyl sulfoxide) for 90 min and then rinsed multiple times and washed for more than 20 min with artificial cerebrospinal fluid (ACSF; 125 mM NaCl, 2.5 mM KCl, 0.9 mM NaH2PO4, 5 mM Na2HPO4, 1.2 mM CaCl2, 1.0 mM MgCl2, 2.5 mM D-glucose). During recordings, the exposed cortex was covered with 50–60 μL ACSF, which was replaced every 20–30 minutes.

### Intracortical stimulation

In one mouse, used to verify electrode locations, stimulation and location marking was performed with a tungsten microelectrode (~200 kOhm resistance, tip diameter ~9 μm; World Precision Instruments, Inc., Sarasota, Florida, USA). Data from this animal were not included in further quantitative analysis. In the other 17 mice, glass microelectrodes filled with ACSF were inserted within a region of 0.5–1 mm anterior from the lambdoid suture and 2.5–3.5 mm lateral from the midline in order to ensure that the electrical stimulation was applied to V1 [37]. The electrodes were bent in an L shape close to the tip. The shaft of the electrode was held horizontally by a micromanipulator (NMN-21; Narishige), enabling movement of the electrode in the confined space between the head of the animal and the object lens and minimizing obstruction of the imaging area, at the same time allowing near-perpendicular insertion of the tip into V1, which helped with the targeting of the stimulation site and depth. The electrodes had a tip diameter of 5–6 μm, resistance ~1 MΩ, and the tip was positioned at a depth of 250 μm below the dura (layer II/III), by first lowering it beyond and then retracting it to the target depth to release possible compression of brain tissue. Anodic-first biphasic current pulses (when not indicated otherwise: intensity 50 μA, phase duration 200 μs, no interphase interval) were delivered with an isolated current generator (STG2008; Multi Channel Systems, Reutlingen, Germany). We found that saturation of the fluorescence response typically occurred beyond 75–100 μA stimulation intensity. In all cases in this study, the stimulation site was at least 500 μm away from the V1/V2 border (in medial, lateral and anterior directions) and the edge of the cranial window (posterior direction), to minimize the effect of these borders on signal propagation. Area borders were estimated based on the cortical activation patterns in the VSD recordings (see “Verification of stimulation site location” in Results).

### Application of GABA_A_ antagonists by electrophoresis

Delivery was performed via glass micropipettes of around 6 μA tip diameter and 1 MΩ resistance filled with either 6 mM bicuculline methiodide (BMI, 2503, Tocris, Bristol, UK) or 1 mM gabazine (GBZ, SR-95531, S106-50mg, Sigma-Aldrich, St. Louis, MO, USA) in saline. A constant 1 μA retaining current was applied in between injection rounds to prevent leakage. The ejection current was 2–5 μA, applied repeatedly in 2–5-minute sessions for both materials.

### Optical imaging and data processing

A fluorescence microscope (THT-microscope; Brainvision, Tokyo, Japan) was set onto the VSD-stained visual cortex, which was illuminated with an excitation light (central wavelength 630 nm). The emitted fluorescence, passing through a dichroic mirror and barrier filter (>665 nm long-pass), was acquired using a CMOS-based imaging system (100×100 pixels; MiCAM-Ultima; Brainvision) for 256–1024 ms at a frame rate of 1 kHz. Acquisition was triggered by the R component of the ECG which made the reduction of vascular pulsation artefacts by subtraction possible. The digitalized data of the fluorescent images were processed on a personal computer. Data calculation and observation were performed with custom-written procedures in MATLAB (The MathWorks, Natick, MA, USA). At each pixel, the mean value of the fluorescence intensity before cortical stimulation (F_0_) and the difference between the fluorescence intensity of each frame and F_0_ (ΔF) were calculated. The relative change in fluorescence (ΔF/F) was calculated by dividing ΔF by F_0_. Each consecutive recording had a lower overall fluorescence level probably due to dye bleaching. In order to eliminate the effects of this on our results, recordings with and without stimulation were taken alternatingly, at ~15 second intervals. The average of the data without stimulation was subtracted from the one with stimulation. When comparing different stimulation intensities, recordings at different intensities were interleaved and ordered to compensate for bleaching (for example in ABC CBA order, where the letters represent different stimulation intensities).

Stimulation at the same current intensity was repeated 10–24 times and these trials were averaged in order to reduce noise. These averages are referred to as trial sets. In most mice, multiple trial sets were acquired at different locations. Quantifications derived from trial sets were first averaged in each animal, and the statistical results in this study are given as the mean ± standard deviation of the averages over animals. A 3×3 or 5×5 pixel Gaussian spatial averaging filter was applied to the data before quantitative analysis. Individual examples shown in figures are all from a trial set. A 3-frames-wide temporal averaging filter was used, if necessary, for video illustrations and time-lapse image sequences only.

### Measurement of latencies

Latencies were measured at absolute or normalized thresholds, depending on the purpose. Thresholds for propagation speeds were normalized to the peak maximum at each pixel to compensate for the changes in the amplitude of the expanding wave. Normalization was not suitable for some of our results, e.g. concerning the falling phase latencies, and in these cases thresholds at absolute ΔF/F levels were used. All thresholds were above the level of statistical significance (2.7 × SD). For comparison we repeated our calculations with a method based on instantaneous phase [38, 39], and found that it yielded similar results to our results with normalized data.

## Results

### Verification of stimulation site location

**Fig 1:**
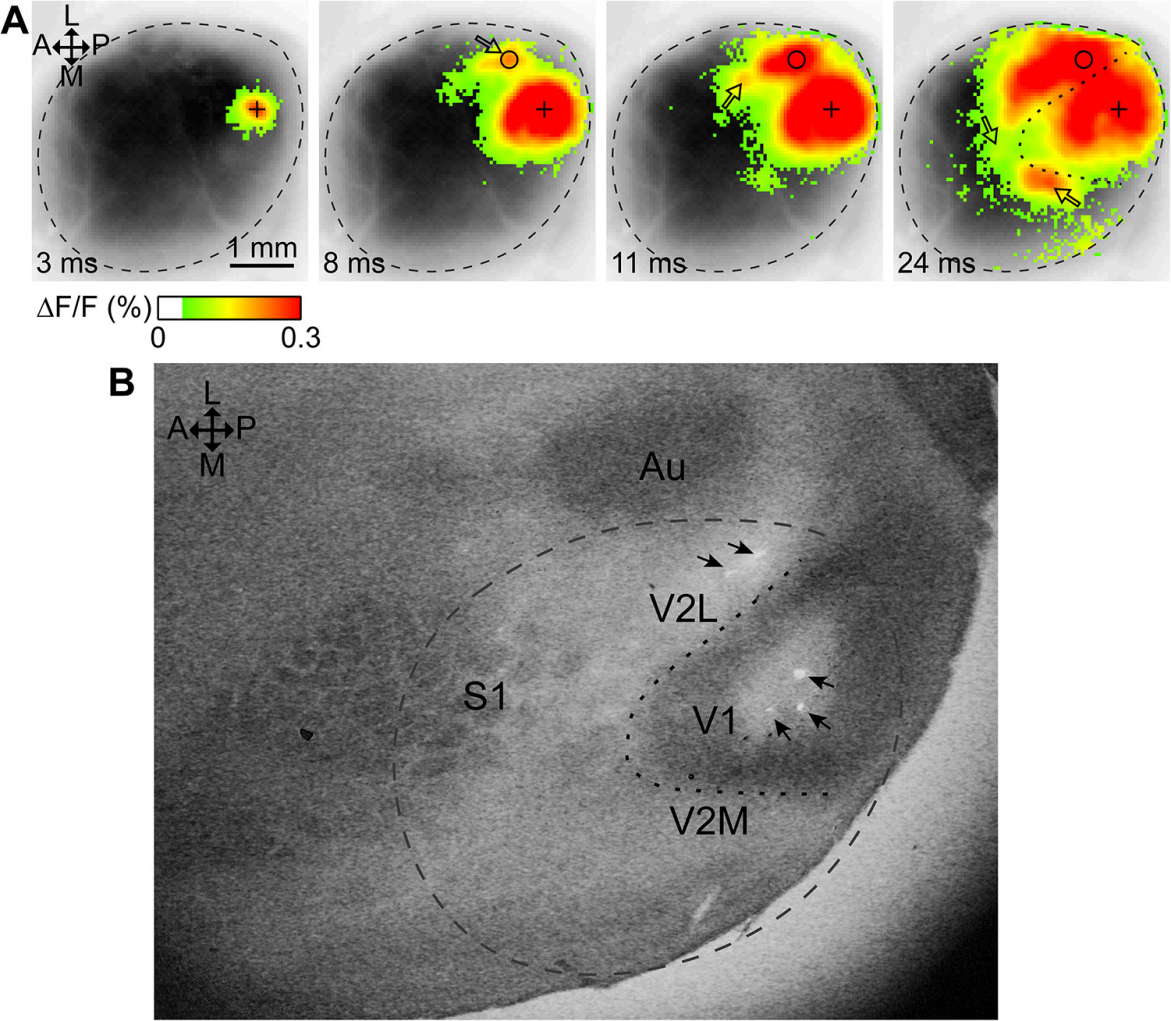
Verification of stimulation site locations. (A) Time-lapse images evoked by a single-pulse 50 μA stimulus in V1 using a tungsten microelectrode (average of 12 trials). ΔF/F values above the level of statistical significance (SD×2.7) are represented in false color as indicated on the bar, superimposed over a greyscale image of the cortical surface. Delay after stimulation indicated on each frame. Stimulation in V1 evoked cortical activity around the stimulation site (cross), and at secondary independent locations (hollow arrows). In the same mouse, 3 stimulation sites in V1 and 2 sites around the first appearing secondary response (circle) were marked by the application of 50 μA for 4 s. (B) Tangential slice of mouse cortex stained for cytochrome oxidase C showing the 5 marked sites as round ruptures in the brain tissue (arrows). The approximate edge of the cranial window, as seen on the VSD image (dashed border) and the approximate border of the heavily stained area (V1) as seen on the slice (dotted curve) are shown in both A and B. The slice and the VSD images were roughly aligned by matching marked sites to the centers of cortical response sites using rotation, scaling and translation. Au: auditory cortex; S1: Somatosensory cortex; V2M: medial V2; A: anterior; L: lateral; P: posterior; M: medial.

Electrical stimulation in V1 induced cortical response indicated by increased fluorescence around the stimulation site, followed by the appearance and expansion of several secondary fluorescence foci lateral, medial and anterior from the stimulation site (Fig 1A). The pattern of these closely matched the spatial pattern of V1 and V2 activity evoked by visual stimulation in mouse [29, 40] and was similar to the anatomical map of mouse visual areas [41], supporting that stimulation in our study targeted V1, and that the independent spot-like activity foci correspond to higher visual areas in the extrastriatal region.

We verified that the assumed V1 stimulation site was indeed within V1 and that the most prominent secondary activity focus was outside of V1 in V2L, by relating VSD responses around stimulation sites in the assumed V1 and V2L areas to marked locations on cytochrome oxidase C (COC) tangential slices (staining method according to [42]) in one animal. First, stimulation was performed at 3 locations in V1, then V2L was stimulated at 2 locations close to the site of the first-appearing secondary response to V1 stimulation. The VSD signal of cortical activity evoked by stimulation was recorded at each site, and then the site was marked by the application of 50 μA current for 3–4 seconds. The three marked V1 stimulation sites are clearly discernible on a post-experimental COC-stained slice from the same mouse within the darker stained striate area (Fig 1B), proving that those stimulation sites were located in V1, as expected. There are two marked stimulation sites lateral to V1, showing that the lateral secondary foci were located extrastriatally. The evoked cortical activity shown on Fig 1A corresponds to stimulation at the most lateral one of the marked V1 sites in Fig 1B.

We aligned the slice with the VSD images as much as possible considering the flattening and shrinkage of cortical tissue during the staining procedure, by matching the 5 stimulation sites on the VSD images to the 5 markings on the slice using translation, scaling and rotation. The location of a stimulation site on the VSD images was defined as the center of the early evoked primary cortical response as expected from the location of the electrode. By superimposing the morphological V1/V2 border in the slice onto the VSD images (dotted line in both panels) we found that it separated the V1 and V2 activity foci, and as such the evoked cortical response pattern is suitable for a rough estimation of the V1/V2 border. For other animals, V1/V2 borders were manually drawn based on the evoked cortical response pattern and activation latency maps. To relate the extrastriatal activity foci to the underlying anatomy of specific visual cortical areas [41], was not in the scope of the present study.

### Time course of fluorescence change

**Fig 2:**
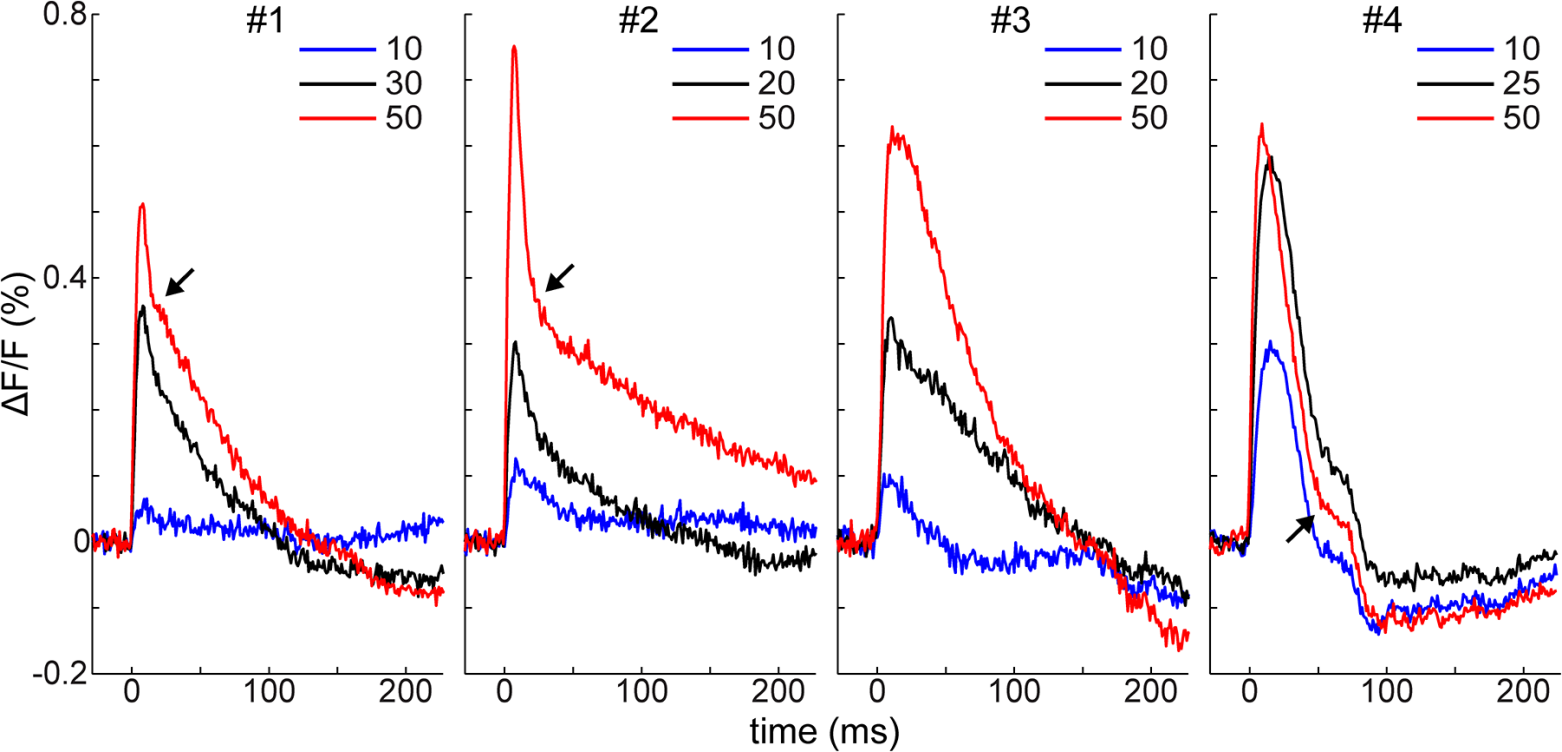
Time courses of evoked VSD response at the stimulation site. Evoked VSD response at 10, 25 and 50 μA stimulation intensities (each an average of 12 trials). Single-pulse electrical stimuli were delivered at 250 μm below the dura with glass micropipette electrodes with 5–6 μm tip diameter and ~1 MΩ resistance. The 4 examples displayed are from different animals. Typically, a fast initial increase of fluorescence was followed by a fast decrease, then a slow decrease or slow undershoot below the baseline. Stimulation at 0 ms; figure legends: stimulation intensity in μA; arrows: break in the downward slope of the signal.

The effect of stimulation was investigated at different intensities in 8 animals. Fig 2 shows examples in 4 of these animals of the cortical response at the stimulation site to weak (10 μA), medium-strength (20–30 μA) and strong (50 μA) stimulation (see S1 Fig for all 8 cases). In case of 50 μA stimulation, the cortical response appeared as a fast-rising transient increase in fluorescence, which was followed by a fast decrease in fluorescence. After the fast decrease, fluorescence levels slowly returned to the baseline (Fig 2, #1–2), or in 4 out of 8 cases it was followed by a slow undershoot below the baseline (#3–4). Furthermore, there was a break or bump in the downward slope of the initial fluorescence peak (#2–4) in 6 out of 8 animals. In some cases (#3–4), the break appeared to separate the fast and slow decreasing phases. Weaker stimulation did not reliably induce cortical responses where these features were distinguishable (with few exceptions, for example #2). We recorded responses at 50 μA stimulation intensity in another 10 (in total 17) mice. The above described basic characteristics (initial transient fluorescent peak, fast and slow decrease) applied to all 17 mice at 50 μA stimulation.

Electrical response to a brief extracellular electrical stimulus recorded in single cells in rat V1 slice is described to consist of an initial EPSP peak followed by fast IPSP that truncates the EPSP peak, and then a slow IPSP [22, 32–34]. The fast and slow IPSPs are believed to be mediated by GABA_A_ and GABA_B_ receptors, respectively [22, 30, 32, 34]. Local VSD signal was found to correlate with subthreshold membrane potential changes in cortical slice preparation [26], and in pyramidal neurons in superficial cortical layers [3], as bath application of the dye strongly stains layers I and II/III [36]. Thus, the VSD signal recorded in our study is expected to reflect the net subthreshold activity of a population of superficial neurons at the recording site. The initial fast-rising fluorescence peak resembled the EPSP peak, which was truncated by a GABA_A_-mediated fast IPSP, similarly to the fast fluorescence decrease we observed. The slow undershoot below the baseline, found in several cases, could indicate the presence of a slow IPSP. For all 50 μA data, the initial EPSP peaked on average at 12.4 ± 6 ms (n = 17, range: 7–26 ms) after stimulation, and the slow undershoot, present in 7 out of 17 animals, peaked on average at 194 ± 79 ms (n = 7, range: 105–296 ms). This can be compared to the 5–10 ms time-to-peak value for the EPSP, and the 157 ± 32 ms peak time for the slow IPSP, reported in [32]. When it is taken into account that the VSD signal is a combined response of a population of neurons, the VSD values can be considered largely in the same range with those reported in single neurons. As shown later, the falling phase of the initial peak was prominently prolonged by application of GABA_A_ synaptic antagonists, which further corroborates the similarity between subthreshold membrane potential changes and the VSD signal.

Action potentials are induced in postsynaptic cells following EPSPs when the intensity of the intracortical extracellular stimulus is several times higher than the threshold intensity [33, 43]. We did not observe transient activity resembling the time course of action potentials in our measurements, even when the current intensity was increased above ~5-fold higher than the threshold intensity. This suggests that action potentials are not discernible in the present measurement as reported in a previous study [3], although many action potentials are activated in synchrony by the high electrical current.

### Spatial pattern of the cortical response

**Fig 3:**
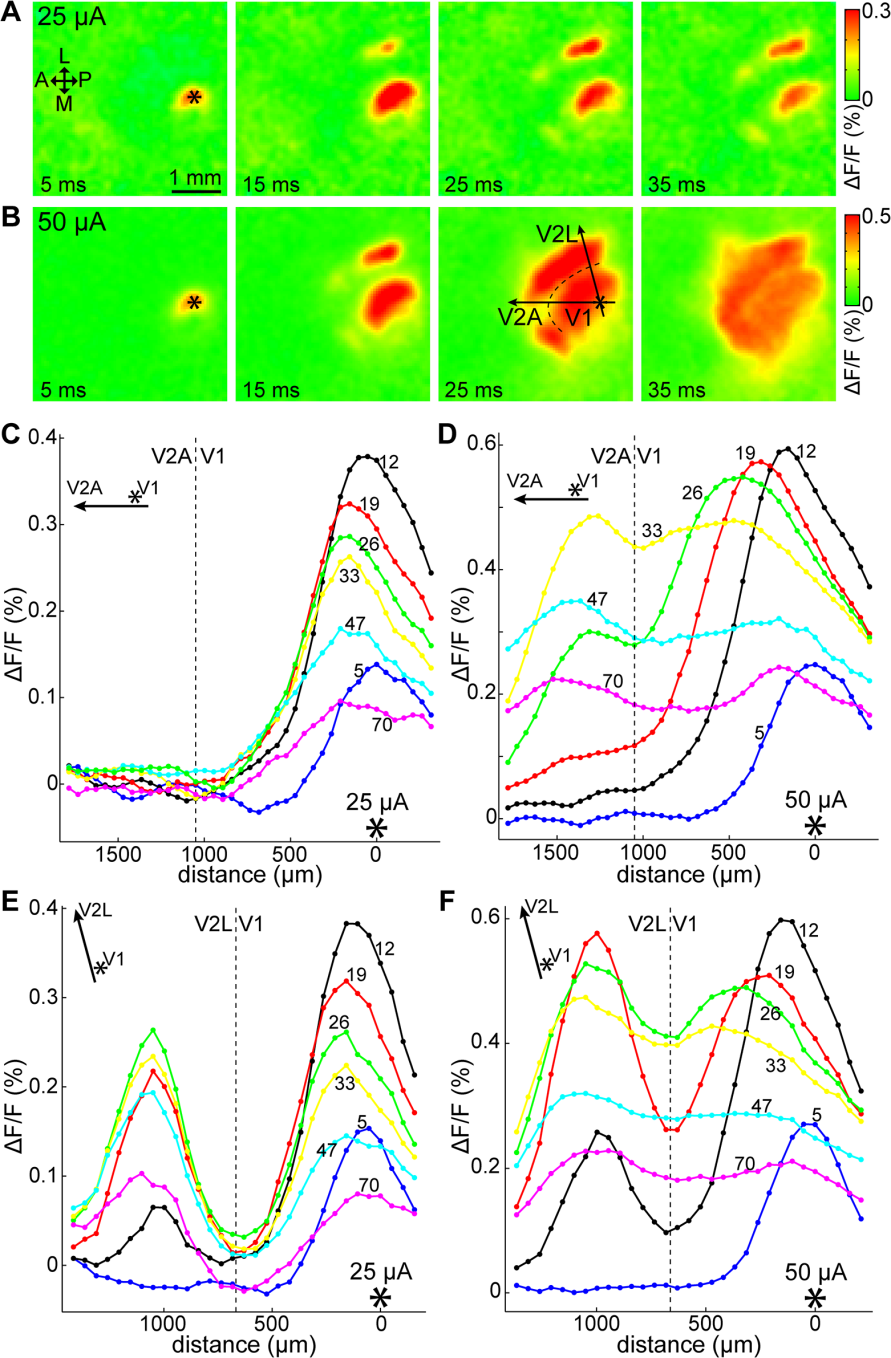
Spatial propagation following high and low intensity stimulation. High-intensity stimulation evoked regenerative propagation in V1. (A, B) Time-lapse images of the VSD signal (averages of 12 trials each) in the right visual cortex evoked by a 25 μA and 50 μA stimulus, respectively, delivered at 250 μm below the brain surface (layer II/III) in V1. Delay after stimulation indicated on each frame. ΔF/F values reproduced as shown on the right bars. A: anterior; P: posterior; L: lateral; M: medial; asterisk: stimulation site. In panel B, 25 ms frame, dashed line: approximate V1/V2 border; arrows: direction of cross-sections in C–F. (C–F) cross-sections of the fluorescence signal along the anteroposterior (C, D) and V1-V2L axes (E, F) (directions indicated by arrows on panel B, 25 ms frame) at increasing delays after stimulation, at 50 μA and 25 μA stimulus, respectively. Stimulus strength and direction indicated on each panel. Delay after stimulation (in ms) shown above each plot. Asterisk: stimulation site; dashed line: approximate V1/V2 border; V2A: anterior V2; V2L: lateral V2.

Fig 3 shows an example of the spatial distribution of VSD signals induced with a single pulse stimulus delivered to V1, at medium-strength (25 μA, Fig 3A) and stronger (50 μA, Fig 3B) intensities in the same animal (also see S1 Video). In both cases, cortical activity became visible around the stimulation site and at several secondary foci in V2. After their initial appearance, the primary and secondary responses expanded radially. In the case of the weaker stimulus, this expansion was spatially limited, whereas in the case of the stronger stimulus, the foci kept expanding, fused together and covered a wide cortical area including V1 and the extrastriatum. This expansion did not stop at the borders of the visual cortex, but spread onto neighboring cortical areas as well. Thereafter, the VSD signals gradually shrunk and diminished. Similar spreading of the VSD signal over cortical boundaries has been previously reported in mouse [44, 45].

The differences are further examined on Fig 3C–3F, which show cross-sections of the fluorescence signal along the anteroposterior and V1–V2L axes (black arrows displayed in Fig 3B, 25 ms frame) at increasing delays after stimulation. Fig 3C and 3D are in anteroposterior direction, whereas 3E-F are aligned along the V1 stimulation site-to-lateral V2 axis. Given that the mouse V1 is spatially more restricted in the mediolateral direction, the anteroposterior axis was found to be more suitable to illustrate V1 cortical activity without being affected by the V1/V2 border, and was preferably used in this and in following figures. There is a striking difference between the two cases: for the high intensity stimulus (Fig 3D and 3F), the peak of the line profile shifted towards anterior (12–26 ms) before it gradually decayed, indicating active horizontal propagation in V1 as opposed to passive diffusion. In contrast, when the stimulus intensity was low (Fig 3C and 3E), there was no extensive horizontal propagation in V1, although the peak shifted slightly toward anterior. Cross-sections in other directions also revealed that the peak shifted away from the stimulation site, suggesting a radially travelling wave. In previous rodent studies, the peak of the VSD signal was reported to occasionally shift in response to electrical [35, 46] and visual stimulation [29].

**Fig 4:**
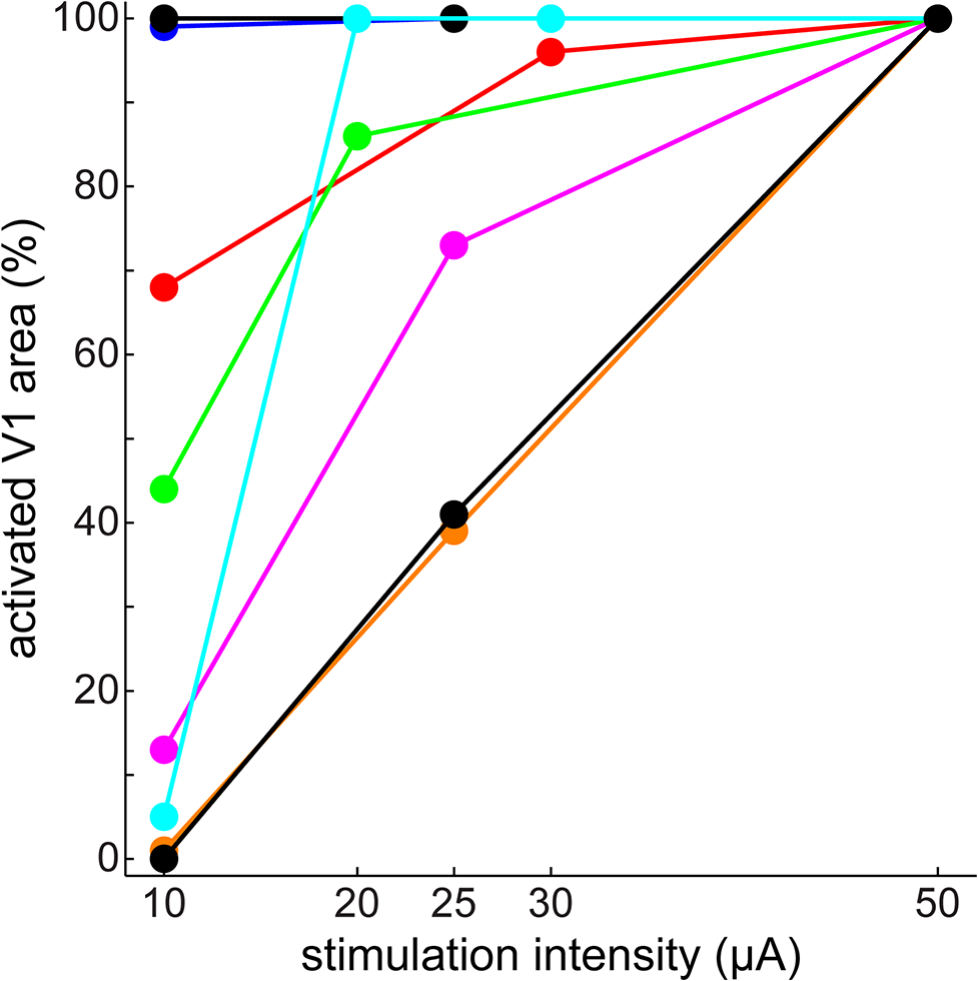
Stronger stimulation is more likely to evoke widespread activation in V1. Activation was defined as fluorescence above the level of statistical significance (defined as SD×2.7) longer than 20 ms at every pixel in V1. Percentage of activated pixels in V1 is shown versus stimulation intensity in 8 animals. Data from each animal is represented with a different color. V1 area maps were drawn manually for each animal based on activation latency maps and activation patterns in the recordings.

In the 8 mice in which different stimulation intensities were compared (the same animals as previously mentioned, see Fig 2), stronger stimulation was likelier than weaker stimulation to cause widespread activation in V1 (Fig 4). Activation was defined as the fluorescence signal staying above SD2.7 noise level for more than 20 ms during a 100 ms period following stimulation. As shown, all stronger stimulation cases induced a widely spreading cortical response. At weaker intensities this was less likely, and in a few cases there was no detectable cortical response. At 50 μA stimulation, we were able to observe widespread activation and also a shift in the peak position as described above in all 17 mice.

### Spatial profile of local VSD response around the stimulation site

**Fig 5:**
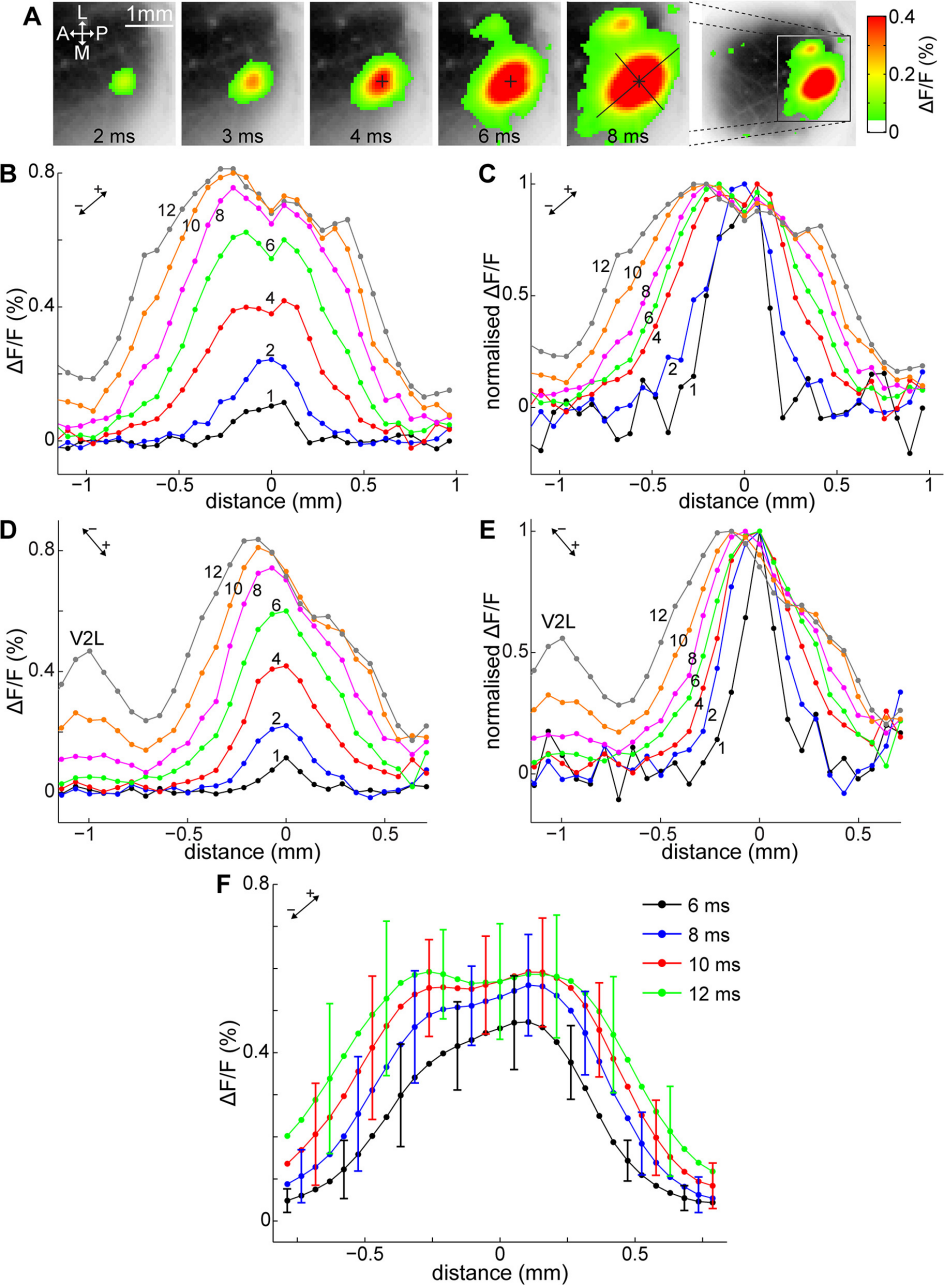
VSD response profile around stimulation site. (A) Time-lapse images of the VSD signal around the stimulation site (cross) in false colors as indicated on the right bar, superimposed over a greyscale image of the cortical surface. Only fluorescence levels above the level of statistical significance (SD×2.7) are displayed. Time after stimulation is indicated on each frame. The cortical response was elongated, the long and short axes of the elongated shape are shown on the 8 ms frame. (B–E) Cross-sections of VSD signal along the long (B, normalized in C) and short axis (panel D, normalized in E) of the cortical response (as shown in panel A, 8 ms), at increasing delays after stimulation. Orientation and direction of the distance axis indicated by inset on each plot. Delay (in ms) is indicated next to each line, and stimulation site is always at 0 distance. V2L: lateral V2. (A–E) are from the same animal, average of 12 trials. Different parts saturated at different ΔF/F levels, indicating that the cause was not VSD response saturation (compare the fluorescence profile on both sides of the stimulation site at 8–12 ms on panel B). (F) Average of cross-sections along the long axis of propagation in 13 mice following 50 μA stimulation. Delays after stimulation are indicated in the legend. Error bars at indicate SD, stimulation site was at 0 distance.

A typical example of the spatial characteristics of early cortical response around the stimulation site following a 50 μA stimulus is shown on Fig 5A–5E. The spatial distribution of the cortical response appeared elongated from a very early stage on (3–4 ms after stimulation; Fig 5A). Cross-sections of the fluorescence signal across the stimulation site are presented in Fig 5B–5E, aligned with the long (Fig 5B, normalized to the maximum value in 5C) and the short axis (Fig 5D, normalized in 5E) of the elongated response area. Fluorescence levels increased after stimulation and saturated within 8–10 ms around the stimulation site (Fig 5B and 5D). After reaching saturation, the area of increased fluorescence continued to expand laterally. As seen in panels B and D, the fluorescence signal exhibited a flattened profile around the stimulation site after the first few milliseconds following stimulation, and dropped quickly outside the flattened region as the distance from the stimulation site increased. The average cross-sections for all 50 μA stimulation cases along the long axis of spreading (Fig 5F) show a similar flattened top, saturation around 10 ms and lateral spreading. To ensure that the flattening and saturation were not an artefact of dye kinetics, only data from those 13 mice have been included into this analysis in which the possible effect of dye saturation could be disproven. Cross-sections in the excluded 4 mice show the same characteristics (see S2 Fig for cross-sections of fluorescence levels in each animal).

As was reported for the activation time course of EPSPs in electrical recordings in rats [22, 32, 33], monosynaptic EPSPs start to be activated with a delay of several milliseconds and reach their maximum amplitude at about 5 ms thereafter in response to a brief electrical shock. Disynaptic EPSPs are not activated in this early period until about 7–8 ms after simulation. Therefore, the line profiles obtained at delays < 7–8 ms after stimulation, comprising an area of ~400–500 μm around the stimulation site (width at half-peak maximum of the 7 ms profile: 841 ± 75 ms, n = 13), mainly reflect the distribution of monosynaptically activated EPSPs. Namely, a brief focal current stimulus applied to the cortex induced monosynaptic EPSPs around the stimulation site, which was followed by disynaptic propagation.

### Propagation velocity and elongation ratio

**Fig 6:**
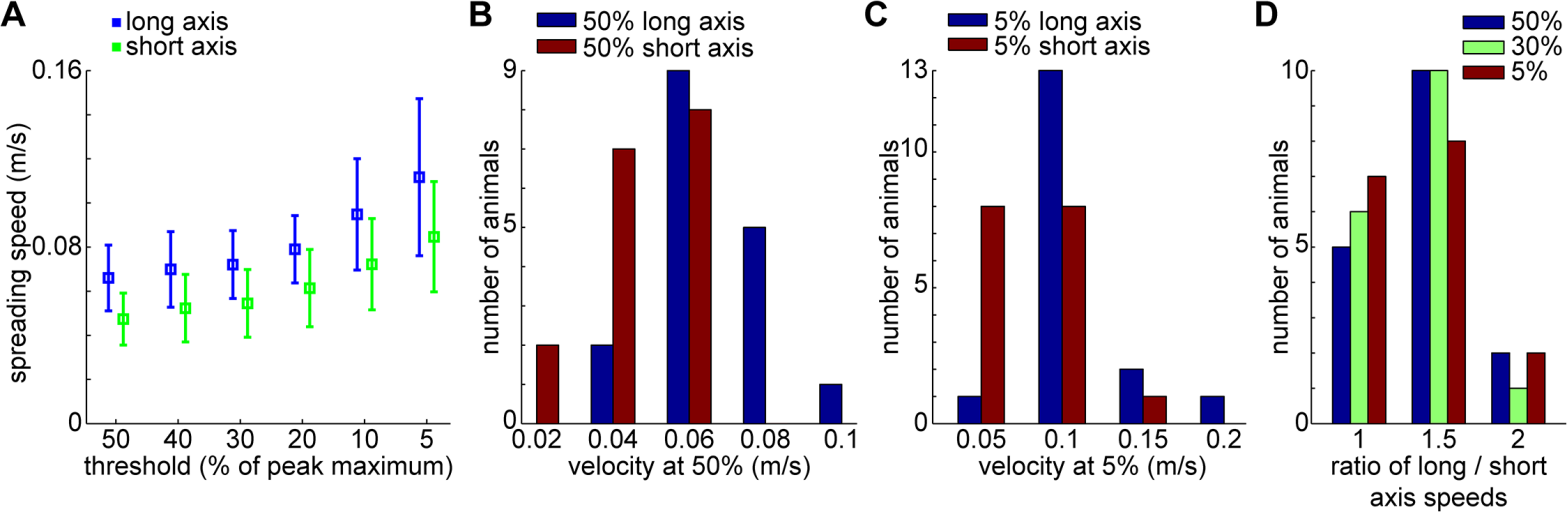
Lateral propagation velocities and anisotropy. Lateral propagation velocities in V1 following 50 μA stimulation at different thresholds, along the long and short axes of the elongated cortical response. Thresholds are relative to peak maximum at each pixel. (A) Means ± SD (n = 17 mice for all panels) of propagation velocities at indicated thresholds. Data points shifted to avoid overlap. (B–C) histograms of spreading velocities at 50% of peak maximum (B) and at 5% (C). (D) Ratio of velocities along the two axes at indicated thresholds. Ratios were calculated for each mouse separately, then averaged. For (B–D), bin centers are indicated, bin widths were equal to the distance between the centers.

Propagation velocity was calculated in the 50 μA stimulation cases as the slope of the line fitted by linear regression on the activation latency values at increasing distances from the stimulation site along the long and short axes of the elongated cortical response. Propagation speed along the long axis was higher at all normalized thresholds than along the short axis (Fig 6A). At half-peak (50%), velocity was 0.066 ± 0.015 m/s along the long axis (n = 17 mice and 50 μA stimulation for all data in this section), and 0.047 ± 0.012 m/s along the perpendicular short axis. At 5% of the peak, velocities were considerably higher, 0.112 ± 0.036 m/s and 0.085 ± 0.025, respectively. The distribution of the spreading velocities at half-peak and 5% levels are indicated in Fig 6B and 6C.

The cortical response propagation was rostrocaudally elongated on average. The long axis of the elongated cortical response was oriented 22.4 ± 9.7° counter-clockwise from the anterior direction, and the average aspect ratio (ratio of velocities) was 1.42 ± 0.23 at half-peak. Fig 6D shows the distribution of the ratios for velocities at 50%, 30% and 5% thresholds. There was no significant difference between the velocity ratios across various thresholds (one-way ANOVA, p>0.05). Based on reference mouse brain coronal slices (Allen Brain Atlas, http://www.brain-map.org/) we estimate that the curvature of the cortical surface can account for ~1–2% of distortion and therefore cannot explain the anisotropy found here. Similar anisotropic propagation was observed in the rat *in vivo* [35]. In anatomical studies, horizontal inter-areal connections were revealed to have a rostrocaudally elongated distribution in rat [19, 23], ferret [47] and in monkey [4], which is the likely underlying reason for the anisotropy found *in vivo*.

### Patterns in the falling phase of the initial fluorescence peak

**Fig 7:**
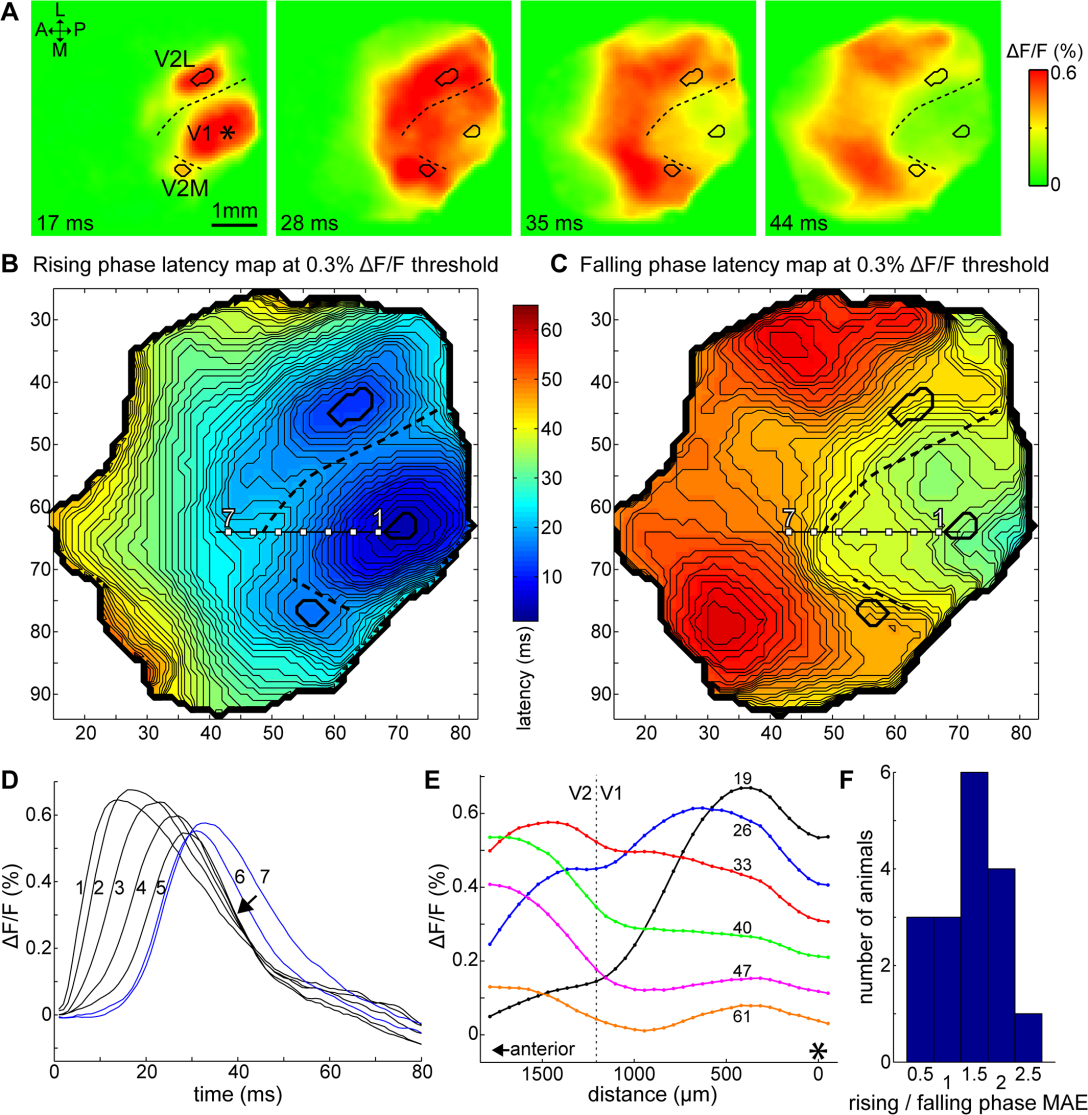
Closely timed falling phases indicate fast-spreading inhibition in V1. (A) Time-lapse images of cortical VSD signal evoked by a single-pulse stimulus in V1. Time after stimulation indicated on each frame. ΔF/F values reproduced as shown in the right bar. Asterisk: stimulation site; V2L: lateral V2; V2M: medial V2; A: anterior; L: lateral; P: posterior; M: medial. (B–C) Latency maps at 0.3% ΔF/F threshold for the rising and falling phases of the initial fluorescence peak. Outlines of the initial responses in V1, V2L and V2M (thick contours) and the estimated functional border between V1 and V2 (dashed curves) were drawn on panels A–C. Latencies represented as shown on the bars. Contour lines were drawn for each millisecond. The very low falling phase latencies found in posterior V1 are calculation artifacts at this threshold due to low signal amplitudes. (D) Time courses of the fluorescence signal at locations 1–7 on panels B and C. Points 1–5 are located in V1; 6 and 7 are in anterior V2 (V2A, blue traces). Arrow: falling phases grouped together. (E) Cross-section of cortical activity along a line segment overlapping locations 1–7 on B and C at increasing delays after stimulation. Asterisk at 0 distance: stimulation site in V1; dotted line: estimated V1/V2 functional border; delay (in ms) indicated next to each profile line. All data in panels A–E are from the same animal, average of 12 trials. (F) Distribution of the ratio of rising and falling phase latency mean absolute errors (MAE) around the median for all 17 mice, between 50–75% of the maximum peak amplitude in V1 for each animal (n = 17, 50 μA stimulation). A >1 ratio indicates that falling phase latencies are more grouped together than the rising phase ones. For each bin, the center is indicated. Bin widths are equal to the distance between centers.

In some experiments, after the initial widespread increase in fluorescence in V1, VSD signals decreased in a pattern that produced a relatively low-fluorescence area with a curved border shaped like the letter C [48]. This is demonstrated in Fig 7A (also see S2 Video). In this example, fluorescence levels started decreasing posterior to and laterally from the stimulation site indicated by an asterisk (28 and 35 ms frames), and a C-shaped border was clearly visible around the low VSD signal area that included the stimulation site (44 ms frame).

The area of this phenomenon roughly matched the area of V1. To investigate this, the spatial distribution of latencies were mapped on the field of view for rising and falling phases of the initial fluorescence peak (Fig 7B and 7C). Contour lines were drawn at every millisecond. The rising phase latency map (Fig 7B) was used to determine response locations around the V1 stimulation site and of independent foci in lateral (V2L) and medial V2 (V2M), and the approximate functional V1/V2 border defined where possible as the area of longest latencies between V1 and V2. These locations (thick contours) and the border (dashed curve) were drawn onto the images and latency maps (Fig 7A–7C) for reference. The falling phase latency map (Fig 7C) clearly shows an area with sparse contour lines around the stimulation site that is surrounded by densely packed contour lines forming a C-like shape. The sparse contour lines indicate that fluorescence levels in that area decreased around the same time (latencies in the majority of V1 fell within a range of 5–6 ms). The borders of this area roughly matched the functional V1/V2 border, confirming that the area of decreased fluorescence corresponds to V1.

By analyzing individual time courses, we found that the rising phases of the initial fluorescence peaks were delayed with increasing distance from the stimulation site, in accordance with lateral propagation, however, the falling phases grouped closely together. This is demonstrated on Fig 7D with time-VSD signal plots sampled at increasing distances anterior from the stimulation site (points 1–7 on the latency maps). Points 1–5 were located in V1, points 6–7 were in anterior V2 (blue traces). More anterior locations had increased delays in the rising phase, but the falling phases banded together (arrow), except for the time course at location 1 that decreased earlier, and time courses from anterior V2. As noted above, the fast falling phase of the fluorescence peak is likely related to the GABA_A_-mediated IPSP that truncates the initial EPSP of cortical neurons. This suggests that a GABA_A_-mediated inhibition appears around the same time all over V1 and suppresses excitation (see also next section).

Fig 7E shows location-VSD signal plots along the same anteroposterior line overlapping points 1–7 taken at increasing delays after stimulation. At 19 and 26 ms after stimulation, horizontal propagation of the cortical activity and the shifting away of the peak from the stimulation site (indicated by an asterisk) are visible. Between 26 and 40 ms, the distribution of the VSD signal flattened in V1, and gradually decreased, which corresponds to the timing of the banding-together of the falling phases on Fig 7D. Anterior V2 (estimated border indicated by the vertical dotted line) VSD signal levels started rising with a delay of ~20 ms after stimulation, and were sustained until after the V1 levels decreased, creating a contrast between these two areas, which appeared as the C-shaped border. This image is similar to Fig 3D and 3F, in another animal, except the evening-out of the V1 activity and the V1/V2 contrast are more easily discernible here. It is noteworthy that the banding of the falling phases and the appearance of the contrasting border between V1 and V2 occur during the fast falling phase of the initial fluorescence peak (around 30–40 ms after stimulation).

With manual inspection of data in 17 mice we found that the VSD signal decreased around the same time over a large area in V1 with a distinguishable C-shaped contrast in 8 animals (S3 Fig). In the rest of the cases, a fast widespread decrease was not clearly discernible in the VSD images. Although these observations are subjective, the contrasting border and fast decrease were present in parts of V1 in several other animals, typically limited to the lateral or anterolateral regions, and falling-phase time courses, when plotted along a line (such as Fig 7D) showed a grouping-together in at least one trial set in each animal. With the following quantitative analysis, however, we found a tendency that the falling phases of the time courses in V1 were more grouped together than the rising phases. We took rising and falling phase latency values at absolute ΔF/F thresholds for all pixels in V1, and calculated the “deviation” of the respective latencies as the mean absolute error (MAE) from the median (1/*n* × Σ|*x*-median|). These statistics were used as they are robust to outliers. Minimum MAE values for the rising and falling phases were selected automatically between 50–75% of the highest peak amplitude in V1, where the grouping-together was generally present. We expected that the rising phase latencies would have a larger MAE, as the cortical activity spreads from the stimulation site, whereas the MAE for the falling phases would be smaller as a result of them grouping together. We found a significant difference between rising and falling phase MAEs (two-sample t-test, p<0.05), with a ratio of 1.4 ± 0.54 on average (rising/falling phase MAE, n = 17 mice, at 50 μA stimulation), >1 in 13 animals (Fig 7F). This ratio is possibly even higher as the calculation method does not take into consideration the slope of the time courses, and as such the “deviation” is more overestimated for the falling phases, which have a slower slope in general.

### Effect of GABA_A_-antagonists on the falling-phase

We investigated the involvement of GABA_A_-mediated inhibitory networks on the falling phase of evoked cortical activity by electrophoretically injecting GABA_A_-antagonists bicuculline methiodide (BMI, 2 mice) and gabazine (GBZ, 4 mice) into the anterior part of V1.

**Fig 8:**
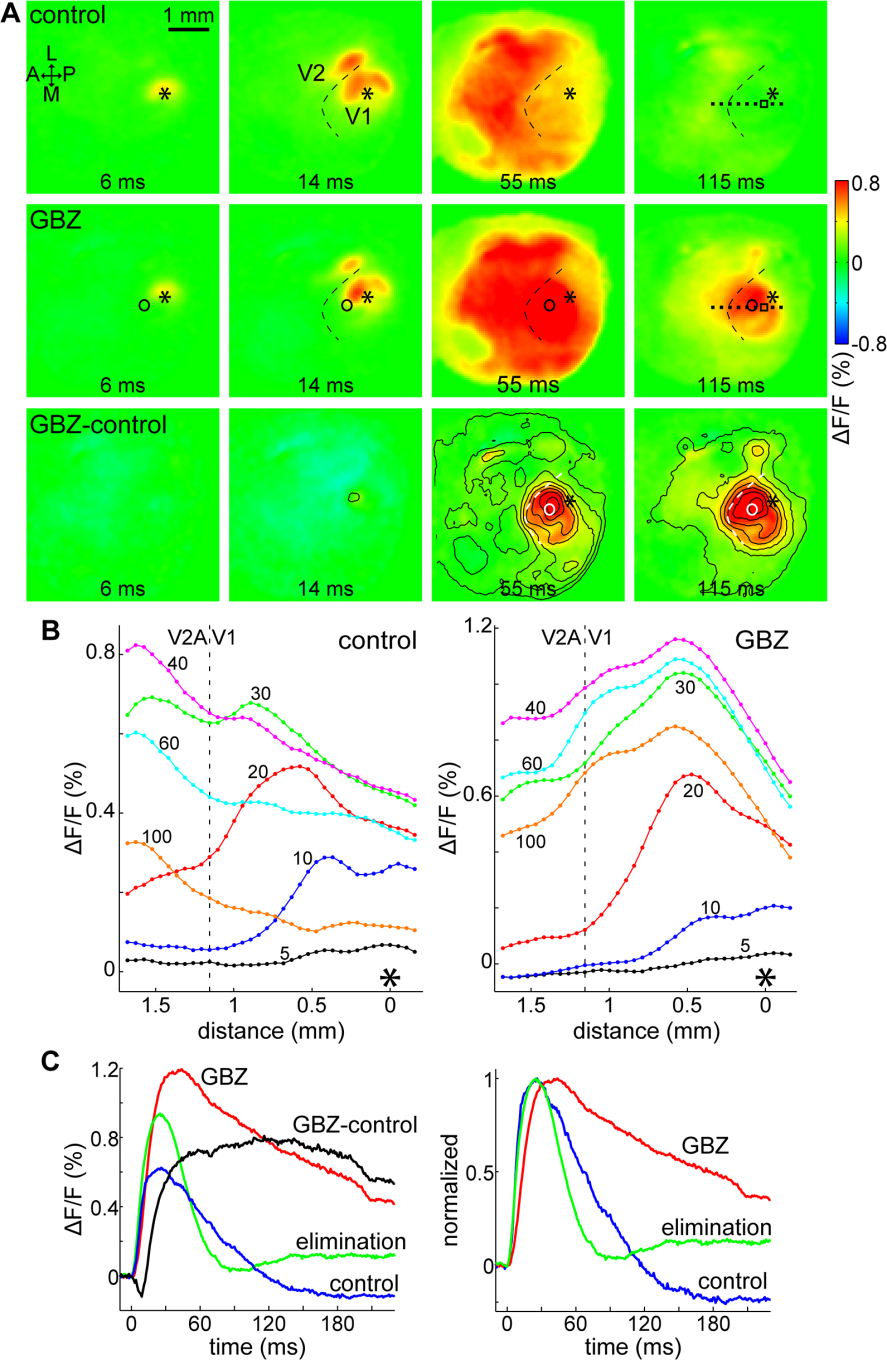
GABA_A_-mediated inhibition strongly affects the deactivation phase in V1. (A) VSD images of evoked cortical activity depicted in color as indicated on the right bar. Control (top row), after focal application of gabazine (GBZ, middle row), and their difference (GBZ-control, bottom row). Frames taken at indicated delays after 50 μA single-pulse stimulation in V1. Contour lines on the difference frames at every 0.1% ΔF/F level above 0. Asterisk: stimulation site; circle: GBZ ejection site; dashed curve: approximate V1/V2 border; A: anterior; L: lateral; P: posterior; M: medial. (B) Cross-section of fluorescence levels in V1 and anterior V2 (V2A) at increasing delays after V1 stimulation. Sampling location indicated by the dotted line segment on the 115 ms frames in panel A. Delays (in ms) are shown next to each line; the dashed vertical line indicates the approximate V1/V2A border, asterisk at 0: position closest to the stimulation site. (C) Time courses of cortical activity in V1 before (control) and after application of gabazine (GBZ), and after ~2 hours elimination. GBZ-control: control subtracted from GBZ. Sampling site indicated by a hollow rectangle between the stimulation and ejection sites on the 115 ms frames in panel A. Left: original data; right: normalized to peak maxima. All data are from the same animal, averages of 16 trials each.

Fig 8A shows an example of evoked cortical activity before and after the application of a GABA_A_ antagonist, in this case GBZ, and the difference of these data (see also S3 Video). The control (top row) and after application of GBZ (middle row) slides show similar activation pattern, however, GBZ eliminated the fast widespread decrease of activity in V1, visible between the borders of V1 (dashed curve) on the control at 55 ms. In addition, the area around the GBZ injection site (circle) remained active longer. The difference between control and GBZ (bottom row) shows that GBZ strongly affected the deactivation phase around the injection site (from the 55 ms frame).

Fig 8B displays the cross-section of the fluorescence signal in V1 and anterior V2 at increasing delays after stimulation along the anteroposterior line segment indicated by the dotted line on the 115 ms frames in panel A. The evening-out of activity levels that was present in the control (left, 60 and 100 ms traces) disappeared after GBZ injection, indicating a change in the falling-phase dynamics. The amplitude of the response inversely depended on the distance from the ejection site, located around 500 μm anterior from the stimulation site, likely due to larger antagonist concentrations towards the center.

The effects of GABA_A_ antagonists on the time course of the VSD signal were quantified in each of the 6 mice for locations in a 10 pixel (~500 μm) radius around the antagonist injection site. Here, significance was calculated with the two-sample t-test, p<0.01, for a dataset including all pixels within the region. The antagonist had a significant (above SD2.7) facilitatory effect starting from 20.8 ± 5.3 ms (n = 6 for every result in this section), around the time that the control time courses reached 60% of the peak (20.1 ± 7.9 ms). Peak amplitude was significantly increased in all cases by 0.64 ± 0.27% ΔF/F, equivalent to an increase of 126 ± 71% on average, and the time-to-half-peak of the falling phase significantly increased in all cases by 68.13 ± 29.7 ms (172 ± 62% increase). The effects of GABA_A_ antagonists were reversed after an elimination time of ~1 hour in case of BMI, and ~2 hours for GBZ. For illustration, see Fig 8C, which shows time courses before and after application of GBZ, their difference (GBZ-control), and after elimination, sampled from a location between the stimulation and injection sites (hollow rectangle on the 115 ms frames on Fig 8A). The time courses on Fig 8C right panel were normalized to their peak maximum. Peak amplitude and duration increased greatly, and the antagonist’s effect (difference curve) was delayed. In this case, early activation was delayed by the antagonist (difference in negative), but this effect was not consistent among animals. As the effects of BMI and GBZ were very similar, no example with BMI is shown to avoid repetition (see S1 Table for individual data). In mouse visual cortex slice, GABA_A_ antagonists similarly did not clearly affect the early phase (11–33 ms), but increased the amplitude and prolonged the duration of VSD responses [28]. These results show that GABA_A_ inhibition had a strong effect starting with a ~20 ms delay after stimulation, which truncated the VSD response peaks, suggesting that GABA_A_-mediated inhibition plays an important part in the fast widespread decrease of cortical activity in V1.

## Discussion

In the present study, we elucidated spatiotemporal properties of wide-range horizontal signal propagation evoked by electrical shocks in mouse V1 in terms of relevant synaptic mechanisms using VSD imaging with a high spatiotemporal resolution (100 x 100 pixels, at ~50 μm / pixel, 1k frames/second). We showed that the time course of cortical activity evoked by direct intracortical electrical stimulation resembled that measured in electrophysiological studies, and that the spatial pattern of activity foci matched that of visual stimulation and known anatomical maps. Basic characteristics of the evoked signal were described: propagation shape and velocity, directly activated region around stimulation site. Lastly, we presented our novel finding that the initial cortical activity peaks were suppressed by a GABA_A_-mediated inhibition that affected all of V1.

The results in this study were based on data in which each animal is represented by a single average value, which was calculated from the trials recorded in each animal. Averaging is beneficial in reducing noise and revealing general tendencies, however, when the data is too diverse, the average may produce artifacts that do not represent underlying cortical behavior. As described in [38], propagating cortical waves can be especially sensitive to averaging and can be missed in trial-averaged data due to variability. Nevertheless, we observed them in both averaged and individual trial data. We compared a subset (411 trials at 50 μA stimulation intensity in 7 mice) of our trial data to the averaged results and found that although there was variation, the overall tendencies (such as expanding widespread activation and the tendency for widespread suppression in V1) were generally present in the majority of the trials: about 10% of the data had very small signal-to-noise ratio, showed large shifts in the baseline or no cortical response; for the rest, ~96% showed widespread expanding activity originating at the stimulation site, and in ~67% suppression in V1 was visible that created a contrast between V1 and V2. Although these measures are subjective, it is clear that the widespread expansion and the widespread suppression in V1 described in this study are not artefacts of averaging. In general, our goal was to characterize overall tendencies in V1 and as such it was not in the scope of this study to discuss individual trials. The elucidation of differences on the trial level remains a task for future research.

Intracortical stimulation is different from visual stimulation, since the dynamics of visually induced responses includes many intervenient processes. Visual stimulation induced cortical activity through the natural pathway, i.e., photo-transduction and projection from LGN. Visually-evoked responses in V1 are slower to appear and last longer (~90 ms latency and ~500 ms duration in [29]; values inferred from figures where no data was indicated), compared with ~10 ms time-to-peak and peak width ~30 ms in case of electrical stimulation. Intracortical stimulation removes the complexity of initial processing steps, and is also able to provide a higher temporal resolution than visual stimulation to study quantitatively how the intra-areal network intrinsic to V1 affects the dynamics of horizontal signal propagation, which allows for a different approach to analyzing cortical circuitry.

Responses to intra-areal electrical shocks have been intensively studied in slice preparations of rodent V1 [22, 23, 30–34], which were used as a reference for our measurements. The VSD signals obtained by us resembled electrically recorded PSPs in these studies. Moreover, the spatial pattern of cortical activity in V1 and in the surrounding visual areas evoked by electrical stimulation (activity foci in lateral and medial V2) was also consistent with that obtained by visual stimulation [29, 40] and was similar to the anatomical layout of mouse visual areas [41], demonstrating that electrical stimulation can be a useful method in the analysis of V1 cortical circuits *in vivo*. The bumps and breaks that we observed on the falling phase of the time courses (Fig 2) in some cases were likely products of inter-areal feedback from V2, whereas in other cases (Fig 2, #1–2) the breaks appeared very similar to those shown in [32] and may have also indicated truncation of the decaying slopes of the EPSPs by fast IPSPs.

Lateral expansion and travelling waves in V1 have been described previously *in vivo* in VSD imaging studies in ferrets [49], rat [46], and recently in mice [29] following visual stimulation. In our experiments, V2 areas became active before the lateral expansion in V1 reached the border (see Fig 3 for an example), thus a wave passing the border could not be discerned, and after the initial fluorescence peak, V1 fluorescence remained at or below the baseline for hundreds of milliseconds. As such, we were not able to observe wave reflection and compression at the V1/V2 border [46].

Tucker and Katz [50] investigated responses to focal electrical train stimulation in tangential slices of ferret visual cortex using VSD. They demonstrated that train stimulation induced numerous ovoid domains as well as a diffuse zone of activation throughout the slice. The ovoid activation domains were consistent with the anatomy of neuronal clusters connected by long-range horizontal connections in rat [18, 19], ferret [51], tree shrews [52], cats [53, 54] and monkeys [55, 56]. VSD imaging on rat visual cortex tangential slices [24] also demonstrated clustered horizontal responses of fast signals, possibly corresponding to the fast transient fluorescence increase in the present study. We did not observe such clustered horizontal responses. This discrepancy could be due to a difference in species or in stimulation method: the current used here could be high enough to have stimulated directly cells belonging to different cluster groups simultaneously, creating the appearance of a continuous response.

In this study, lateral spreading velocities ranged from 0.08–0.15 m/s at 5% of peak maximum to 0.05–0.08 m/s at half-peak maximum, in the direction of fastest spreading. This can also be seen on the time courses: fluorescence levels start rising very early even far from the stimulation site (Fig 7D, see the start of rising phases at locations 1–5). In the later part of the rising phase, however, the slope gradients are similar and the time courses are further apart. The fastest spreading speeds are within the range of lateral propagation velocities measured in slice and *in vivo* studies in rodents [22], ferret [47, 50], cat [2, 43] and monkey [4], which are considered to be mediated by monosynaptic long horizontal connections. The slow spreading was comparable to propagation speeds in rat visual cortex slices measured with VSD (~0.06 m/s; [21]).

Electrical stimulation activates cell processes around the tip, which could cause the activation of neurons millimeters away. This is because cell projections passing near the electrode tip are activated, which could have targets close or far away [57]. Activated long-range connections, which require one or a few synapses to reach an area, could be responsible for the observed faster propagation speeds. Long-range connections have been extensively described in previous studies [2, 4, 22, 43, 47, 50]. Propagation through shorter-range connections, similar to neighbor-to-neighbor short horizontal connections reported in [58], is slower as it requires multiple synapses. Repeated activation of subsequent neighboring cells in such a multisynaptic network could create regenerative propagation that reaches all over V1. In our observation, the dominant part of the VSD signal propagated slowly outward from the stimulation site, suggesting that short-range connections may be more numerous than long-range ones. High stimulus intensities (50 μA, used in most of our experiments) induced lateral expansion that covered all of V1. At lower stimulation intensities, lateral spreading of the excitatory signal was often confined to a small area around the stimulation site. The reason for this could be that the stronger electrical shocks, besides activating long horizontal projections, by stimulating a large number of neurons around the electrode tip simultaneously and synchronizing their spiking, were able to effectively induce a regenerative wave across multiple synapses through shorter lateral connections, while the weaker stimulus was less likely to activate a sufficient number of neurons to induce a regenerative travelling wave, and the activity transmitted through directly activated (non-regenerative) activity, for example through long-range connections, was below detection threshold. Several alternative explanations are possible for the slow propagation: local cortical dynamics and feedback from LGN or V2. Local dynamics could influence integration time, such as feedforward inhibition [59]. Such a mechanism cannot be completely excluded with our methods, but in our recordings the application of GABA_A_-antagonists did not clearly affect early rising-phase propagation. In fact, absolute latencies increased slightly in 4 out of 6 mice (15 ± 25%, range -1.6–3.8 ms, n = 6 at 0.3 ΔF/F) in the presence of GABA_A_ antagonists. Other candidates for the origin of slow spreading could be feedback from LGN and V2 areas. To gauge their possible effect, feedback latencies will be estimated using the shortest excitatory-neurons-only loop comprising two synapses (0.5–1 ms synaptic delay each) as a model. Slow propagation (at half-peak maximum) is already present as early as 6–8 ms after stimulation, therefore if these areas are the main origin of such propagation, feedback latencies should be shorter than this value. LGN-to-visual cortex latency is around 5 ms (4.9 ± 0.5 ms [60]), so the feedback loop would take around 11 ms (9.8–12.8 ms) to complete at the earliest. Feedback latency from V2 depends on interareal conduction velocity and distance. With V1-LM feedforward and feedback conduction velocities around 0.35–0.4 m/s [61] and a distance of 1.7 mm (we observed slow propagation in multiple cases stimulating at this distance from LM), corresponding feedback latencies would be 9.5–10.7 ms. In all our data, other V2 areas activate after LM thus, assuming similar conduction velocity and distance, they would have longer latencies. These estimates suggest that interareal feedback is unlikely to be the exclusive origin of slow propagation, however, further investigation is needed to reveal the transmission mechanism more accurately. Our results suggest that besides fast-transmitting long projections, slow transmission through multiple synapses could also play an important part in horizontal propagation, and consequently in integrative functions in V1.

In accordance with the propagation pattern of the VSD signal, the rising phase showed a latency that increased with distance from the stimulation site. Interestingly, however, the falling phases of the time courses from a large part of V1 were grouped together, as shown on Fig 7. This suggested a fast-spreading inhibition that covered all of V1. This coordinated suppression was removed by the application of GABA_A_-antagonists gabazine and bicuculline methiodide, indicating that GABA_A_-mediated inhibition was strongly involved in the truncation and synchronization of the falling phase. In most of our data the grouping-together of falling-phase time courses was already present at 30–40 ms after stimulation, which is too early to be affected by GABA_B_ (onset estimated at 50 ms, based on figures in [22, 30, 32, 34]). Although such large-scale inhibition may not be present in the same way under more natural circumstances (visual stimulation, for instance), the existence of a wide-ranging inhibitory network could be relevant to non-classical RF functions in higher mammals [10, 13–15, 17, 62]. The short time range in which the suppression appears in V1 is unlikely to be explained merely with intra-areal circuits that are limited to axonal conduction velocities–a more viable explanation is feedback from higher visual areas, LGN or a combination of these mechanisms.

Anesthesia has been criticized in recent studies for altering the characteristics of cortical activity as compared to an awake state. Although somatosensory-evoked responses in the rat and mouse were reported to have a larger amplitude and activated area in awake state, the signals were qualitatively similar between states [63, 64]. These results may not be applicable to our case as we used direct intracortical stimuli and some of the reported differences may not be intrinsic to the visual cortex. A recent study comparing several sensory-evoked responses, including visual stimuli, in awake and anesthetized mice describes higher peak amplitude and longer decay times in awake mice, but otherwise similar responses in both conditions [65]. Direct optogenetic activation produced haemodynamic responses similar to those in awake state [66]. On a microscopic scale, anesthesia decreased suppression strength in parvalbumin-expressing interneurons [67] and caused changes in synchronization in local networks [68], but it is difficult to interpolate these may affect cortical activity on a larger mesoscopic scale. While widespread propagating waves are generally present under anesthesia, such as in our case, their existence in awake animals was not clear. Polack and Contreras [29] reported that, in mice under neuroleptanalgesia and visual stimulation, in 61% of the cases the responses did not propagate, and only in 23% of the trials did the response spread beyond the boundaries of V1. However, Muller et al. [38] observed the consistent presence of widespread propagating waves in awake monkey V1 evoked by visual stimuli, which supports our results. We believe, therefore, that above differences do not invalidate our findings.

In summary, we showed that direct cortical electrical stimulation induced VSD signals with a time course similar to EPSP and IPSP in electrophysiological measurements. Evoked cortical activity appeared in V1 and other visual areas (in accordance with known mouse visual cortex anatomy), and expanded radially to cover V1 in the form of a travelling wave. The expansion was elongated rostrocaudally, as expected from the anatomy of horizontal projections., and spreading velocities suggested combined faster and slower transmission, likely via connections ranging from long-range fast, possibly monosynaptic or oligosynaptic, to slower short-range multisynaptic. Following the transient activity peak, a widespread GABA_A_-mediated inhibition suppressed VSD signals in V1 around the same time. These results are the first quantitative analysis of cortical activity evoked by direct electrical stimulation in mouse visual cortex, and help us understand the neuronal circuitry involved in large-scale visual processing functions in V1.

## Supporting Information

S1 Fig Comparison of the effect of different stimulation intensities. Time courses of the fluorescence signal at the stimulation site following low, medium and high intensity stimulation. This figure complements Fig 2: data for all 8 animals in which stimulation intensities were compared are shown here. Each time course is the average of 10–16 trials, from top left, respectively. Legends indicate stimulation intensities (in μA). Axis labels are the same for each panel.
(TIF)

S2 Fig Spatial pattern of local spreading around the stimulation site in all 17 mice. This figure complements Fig 5: cross-sections of the fluorescence signal around the stimulation site are shown for all 17 animals here. Each panel, one per animal, represents the averaged data from all trial sets taken at 50 μA stimulation intensity in the same animal. The cross-sections are oriented along the long axis of spreading. Graph axis labels, indicated in the first panel, are the same for all panels. Colors correspond to delays (in ms after stimulation) as indicated in the legend. Data with red frames have been excluded from this analysis (including Fig 5F) because a possible effect of dye saturation could not be disproven. They are shown here to demonstrate that flattening and saturation was present in all animals.
(TIF)

S3 Fig Further examples of widespread suppression of the fluorescence signal. This figure complements Fig 7: false-color images are shown from all 8 animals where the overall decrease of V1 fluorescence levels created a discernible contrasting border between V1 and V2. Estimated V1/V2 borders, first appearing V2L and V2M activity foci (manually drawn based on latency maps and activation pattern), and delay after stimulation are indicated on each image. Each image represents data from a different animal, and stimulation intensity was 50 μA in all cases. Ant: anterior; Post: posterior; Lat: lateral; Med: medial.
(TIF)

S1 Table This table contains the data underlying the statistical results and figures. Data present for each animal where applicable.
(XLSX)

S1 Video Comparison of 25 μA and 50 μA stimulation. The cortical responses induced by stimulation at 25 μA (left) and 50 μA (right) intensities are shown side-by-side. Color coding as shown on the color bars in the video. Delay after stimulation (in ms) is shown under the frames. Same data as in Fig 3.
(MP4)

S2 Video Suppression of the fluorescence signal in V1. Stimulation in V1 at 50 μA induced a widespread cortical response that was followed (from around 34 ms) by the overall decrease of the fluorescence signal in V1, creating a contrast between V1 and V2. The data is from the same animal as in Fig 7. The VSD signal is represented in the colors indicated on the color bar; only statistically significant activity (> SD×2.7) is displayed. Delay after stimulation (in ms) is indicated under the frame in the video.
(MP4)

S3 Video Effects of gabazine. This video complements Fig 8: control, after gabazine (GBZ) and difference (GBZ-control) recordings are compared side-by-side. The data is from the same animal as in Fig 8. The VSD signal is represented in the colors indicated on the color bar. Delay after stimulation (in ms) is indicated under the frame in the video.
(MP4)
