# Supplementary figures and images for: In Vivo Voltage-Sensitive Dye Study of Lateral Spreading of Cortical Activity in Mouse Primary Visual Cortex Induced by a Current Impulse
Source: PLoS One. 2015 Jul 31;10(7):e0133853. doi: 10.1371/journal.pone.0133853 (PMC4521781; doi:10.1371/journal.pone.0133853)

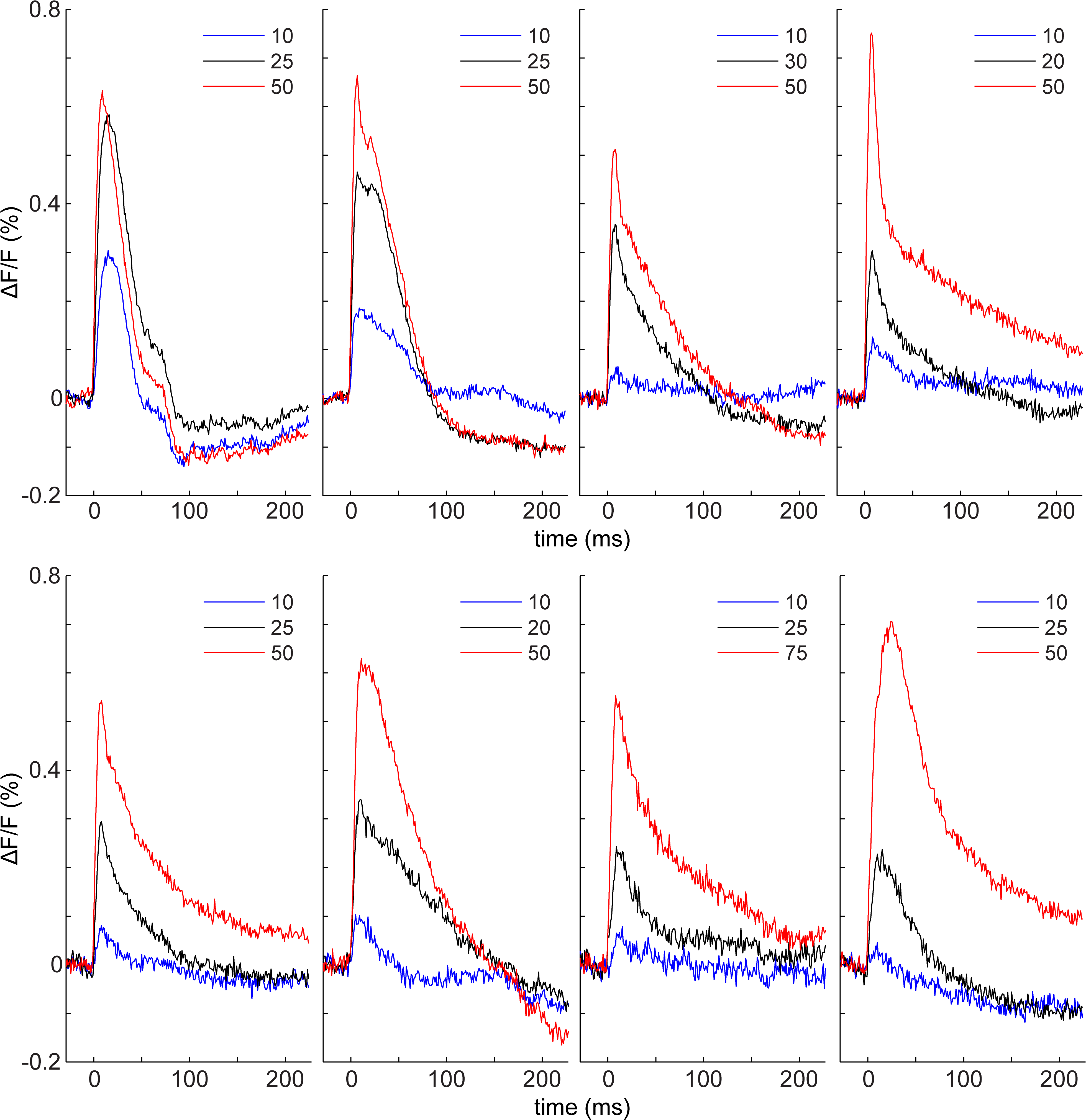

Supplement: S1 Fig — Time courses of the fluorescence signal at the stimulation site following low, medium and high intensity stimulation. This figure complements Fig 2: data for all 8 animals in which stimulation intensities were compared are shown here. Each time course is the average of 10–16 trials, from top left, respectively. Legends indicate stimulation intensities (in μA). Axis labels are the same for each panel. (TIF) [file pone.0133853.s001.tif]

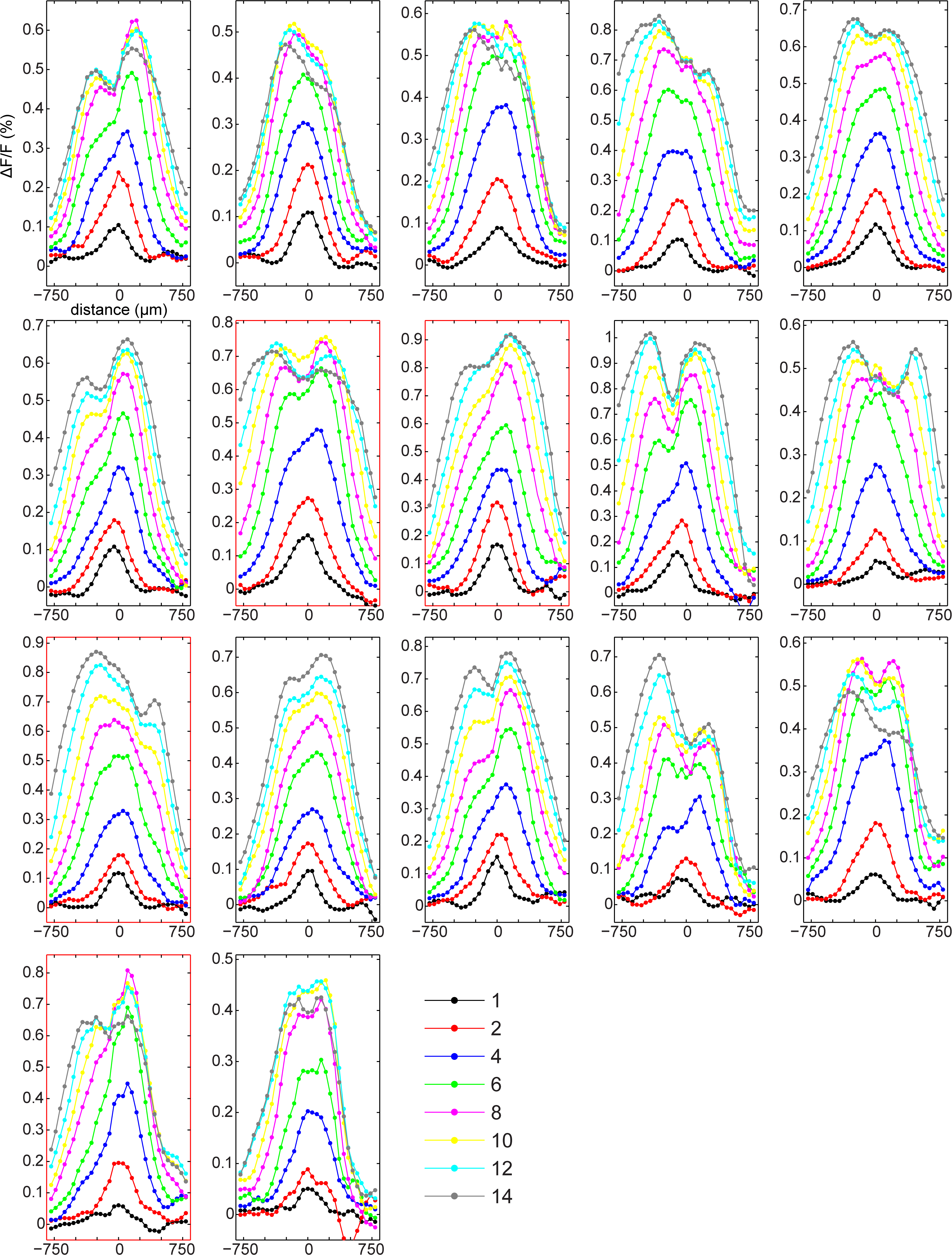

Supplement: S2 Fig — This figure complements Fig 5: cross-sections of the fluorescence signal around the stimulation site are shown for all 17 animals here. Each panel, one per animal, represents the averaged data from all trial sets taken at 50 μA stimulation intensity in the same animal. The cross-sections are oriented along the long axis of spreading. Graph axis labels, indicated in the first panel, are the same for all panels. Colors correspond to delays (in ms after stimulation) as indicated in the legend. Data with red frames have been excluded from this analysis (including Fig 5F) because a possible effect of dye saturation could not be disproven. They are shown here to demonstrate that flattening and saturation was present in all animals. (TIF) [file pone.0133853.s002.tif]

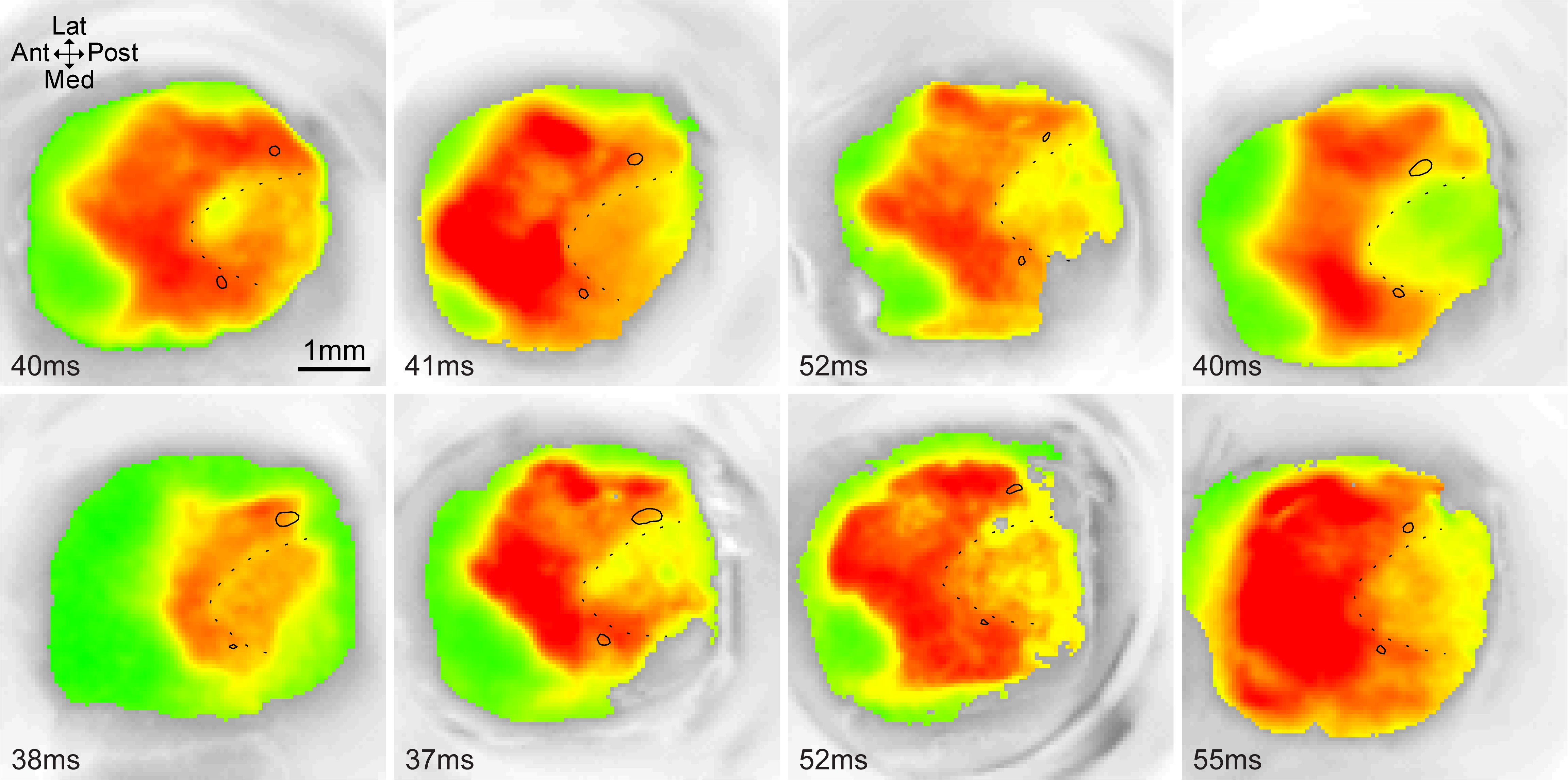

Supplement: S3 Fig — This figure complements Fig 7: false-color images are shown from all 8 animals where the overall decrease of V1 fluorescence levels created a discernible contrasting border between V1 and V2. Estimated V1/V2 borders, first appearing V2L and V2M activity foci (manually drawn based on latency maps and activation pattern), and delay after stimulation are indicated on each image. Each image represents data from a different animal, and stimulation intensity was 50 μA in all cases. Ant: anterior; Post: posterior; Lat: lateral; Med: medial. (TIF) [file pone.0133853.s003.tif]
